# Cost-Effectiveness Analysis of Combination Therapies for Visceral Leishmaniasis in the Indian Subcontinent

**DOI:** 10.1371/journal.pntd.0000818

**Published:** 2010-09-07

**Authors:** Filip Meheus, Manica Balasegaram, Piero Olliaro, Shyam Sundar, Suman Rijal, Md. Abul Faiz, Marleen Boelaert

**Affiliations:** 1 Department of Public Health, Institute of Tropical Medicine, Antwerp, Belgium; 2 Drugs for Neglected Diseases Initiative, Geneva, Switzerland; 3 United Nations Children's Fund (UNICEF)/United Nations Development Programme (UNDP)/World Bank/World Health Organization (WHO) Special Programme for Research and Training in Tropical Diseases, World Health Organization, Geneva, Switzerland; 4 Nuffield Department of Medicine,The Centre for Tropical Medicine, Centre for Tropical Medicine and Vaccinology, University of Oxford, Churchill Hospital, Oxford, United Kingdom; 5 Institute of Medical Sciences, Banaras Hindu University, Varanasi, India; 6 B.P. Koirala Institute of Medical Sciences, Dharan, Nepal; 7 Sir Salimullah Medical College, Mitford, Dhaka, Bangladesh; Texas A&M University, United States of America

## Abstract

**Background:**

Visceral leishmaniasis is a systemic parasitic disease that is fatal unless treated. We assessed the cost and cost-effectiveness of alternative strategies for the treatment of visceral leishmaniasis in the Indian subcontinent. In particular we examined whether combination therapies are a cost-effective alternative compared to monotherapies.

**Methods and Findings:**

We assessed the cost-effectiveness of all possible mono- and combination therapies for the treatment of visceral leishmaniasis in the Indian subcontinent (India, Nepal and Bangladesh) from a societal perspective using a decision analytical model based on a decision tree. Primary data collected in each country was combined with data from the literature and an expert poll (Delphi method). The cost per patient treated and average and incremental cost-effectiveness ratios expressed as cost per death averted were calculated. Extensive sensitivity analysis was done to evaluate the robustness of our estimations and conclusions. With a cost of US$92 per death averted, the combination miltefosine-paromomycin was the most cost-effective treatment strategy. The next best alternative was a combination of liposomal amphotericin B with paromomycin with an incremental cost-effectiveness of $652 per death averted. All other strategies were dominated with the exception of a single dose of 10mg per kg of liposomal amphotericin B. While strategies based on liposomal amphotericin B (AmBisome) were found to be the most effective, its current drug cost of US$20 per vial resulted in a higher average cost-effectiveness. Sensitivity analysis showed the conclusion to be robust to variations in the input parameters over their plausible range.

**Conclusions:**

Combination treatments are a cost-effective alternative to current monotherapy for VL. Given their expected impact on the emergence of drug resistance, a switch to combination therapy should be considered once final results from clinical trials are available.

## Author Summary

Visceral leishmaniasis (VL) is a serious health problem in the Indian subcontinent affecting the rural poor. It has a significant economic impact on concerned households. The development of drug resistance is a major problem and threatens control efforts under the VL elimination initiative. With an unprecedented choice of antileishmanial drugs (but no newer compound in clinical development), policies that protect these drugs against the emergence of resistance are required. A possible strategy that has been successfully used for malaria and tuberculosis is the use of combination therapies. This study is the first comprehensive assessment of the cost-effectiveness of all possible mono- and combination therapies for the treatment of visceral leishmaniasis in the Indian subcontinent. The analysis was done from the societal perspective, including both health provider and household costs. The present work shows that combination treatments are a cost-effective alternative to current monotherapy for VL. Given their expected impact on emergence of drug resistance, the use of combination therapy should be considered in the context of the VL elimination programme in the Indian subcontinent.

## Introduction

Despite their toxicity, pentavalent antimonials are still widely used as first line treatment for visceral leishmaniasis (VL) except in the Indian subcontinent where emerging drug resistance in Bihar State in India [1] and Nepal [2] required a change in drug policy. Current therapeutic options include amphotericin B deoxycholate (AmB), liposomal amphotericin B (L-AmB), miltefosine (MF) and paromomycin (PM). The VL elimination initiative launched in 2005 by the governments of India, Nepal and Bangladesh adopted miltefosine as the first line treatment [3], [4]. More recently paromomycin was registered in India as a first line regimen for VL [5]. However, parasite resistance to MF and PM can be induced experimentally [6] and is expected to emerge naturally if optimal adherence cannot be ensured [7]. The World Health Organization has recommended the use of liposomal amphotericin B (L-AmB) based on high efficacy and safety [8].While the development of resistance has not yet been demonstrated for AmB and L-AmB, practicalities (requirements for cold chain and intravenous perfusion) and the high drug cost have so far delayed its adoption as first line treatment. As there are no new compounds for VL expected to come to the market in the near future, policies that delay or prevent the emergence of resistance to the currently available drugs are therefore required. A possible strategy that has been successfully used for malaria and tuberculosis is the use of combination therapies [9]. Combination therapies may also increase tolerability, reduce treatment duration and possibly (direct and indirect) costs.

Phase III clinical trials of combination therapies for VL are currently underway testing the efficacy and safety of several combinations and results are expected in 2010 (Clinicaltrial.gov, Identifier: NCT00696969; for more information see http://clinicaltrials.gov/). Choices in VL drug policy should be based on efficacy, safety as well as the cost of treatment, the process of patient management and the factors influencing treatment effectiveness, such as adherence. The latter factor is particularly important as some regimens (e.g.injectables) are likely to lead to higher compliance than others.

The objective of the present study was to assess the cost-effectiveness of various treatment options for VL, and in particular to evaluate whether combination therapies are a cost-effective alternative to monotherapy.

## Methods

### Description of alternatives

We considered 10 alternative treatment strategies: (1) all monotherapies that are either already implemented or under consideration and (2) combination therapies currently included in a phase III clinical trial (See table 1). AmB has infusion-related and delayed toxicities (e.g.nephrotoxicity) [10] and requires prolonged parenteral administration and hospitalisation. MF has the advantages of an oral drug but causes serious adverse events in 2–3% of patients [11] , has a long half-life and is possibly teratogenic. It can thus not be used in pregnant women and women in child-bearing age should accept contraception over the treatment period and up to two months after [12]. PM seems a safer option - though phase IV results are still pending - and relatively cheap, but requires intramuscular injections. L-AmB is highly efficacious (>90%) even in a single dose of 5–10 mg/kg in India [13]–[15] and is safe, but it is expensive despite a preferential price offered by the manufacturer to the public sector and requires an efficient cold chain. All of the other more “affordable” monotherapies listed above (MF, PM, AmB) require prolonged treatment which is problematic in very poor population groups that are dependent on daily labour and pay much out-of-pocket.

**Table 1:** Overview of treatment strategies included in decision analysis model.

Strategy	Drug	Length of treatment (days)
A	L-AmB (5MK)+Miltefosine (50/100 MD)	8
B	L-AmB (5MK)+Paromomycin sulphate (15 MKD)	11
C	Miltefosine (50/100 MD)+Paromomycin sulphate (15 MKD)	10
D	SSG (20 MKD)+Paromomycin sulphate (15 MKD)	17
E	Miltefosine (50/100 MD)	28
F	Paromomycin sulphate (15 MKD)	21
G	Amphotericine B deoxycholate (1 MK eod)	30
H	L-AmB10 (10 MK)	1
I	L-AmB20 (5 MKD)	4
J	Sodium Stibogluconate (20 MKD)	30

- L-AmB : Liposomal Amphotericine B.

- MK = mg/kg single dose; MD = mg per day; MKD = mg/kg body weight per day.

- Miltefosine is given at 50 mg/day if body weight is <25 or 100 mg if body weight ≥25 kg per day.

### Decision model

**Figure 1:**
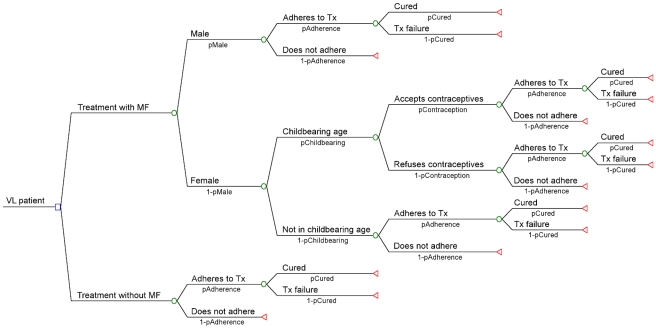
Root decision tree with different pathways depending on whether miltefosine is included in the strategy.

A decision tree model, depicted in figure 1, was developed using TreeAge Pro Suite v2009 (TreeAge Software Inc., Williamstown, MA, USA) to determine the outcome of a single confirmed VL patient receiving first-line treatment at a primary health care facility. The outcome was expressed in terms of number of deaths averted and we assumed a case-fatality rate of 100% in the absence of treatment. For each treatment strategy, the patient either adheres or does not. Those adhering are either cured or experience treatment failure. Patients not adhering to treatment were considered lost to follow-up and we assigned a value of 0 deaths averted. For strategies combining L-AmB with MF or PM, we assigned a value of 0.91 in case of non-adherence since patients will have received a single dose of 5mg/kg of L-AmB (with 91% cure rate) [16], [17] on the first day before they are lost to follow-up. Since MF is contraindicated in pregnant and breastfeeding women or women in child bearing age because of its potential teratogenic effect, the path for strategies including MF is different from those without MF. MF can only be given if the patient accepts the use of contraceptive measures during treatment and up to two months after completion of treatment. This is captured in the model by including an additional probability representing the contraceptive prevalence in the community. We calculated the cost per case treated and the average and incremental cost-effectiveness ratios (ICERs) expressed as the cost per death averted. The ICER represents the additional cost to gain an additional unit of effectiveness (i.e. one additional death averted) and is calculated by dividing the incremental cost of a given strategy by its incremental effectiveness compared to the previous not dominated strategy.

Furthermore we assumed in the baseline analysis the patient to be hospitalized for at least one day for all strategies; for treatment with AmB the patient is hospitalized for the entire duration of treatment (30 days) as this drug needs to be given under close supervision. Strategies with PM and SSG are provided on an outpatient basis whereby the patient visits the health facility daily to receive the intramuscular injection. In the case of treatment with MF, the patient visits the health facility weekly to receive a 1-week supply of the drug. We also assumed patients to undergo weekly routine laboratory investigations (blood count, liver and renal function tests), a pregnancy test for women in child-bearing age and an HIV test.

### Probabilities

The probabilities used in the model are shown in table 2. These consist of a most plausible value used in the baseline analysis and the range used in the sensitivity analysis. We used anthropometric data from a sample of 1496 patients attending a dedicated VL treatment centre in Muzaffarpur, Bihar (India) [18] to derive probabilities on patient characteristics (age, weight and sex). Other values were obtained from expert opinion and published literature. To derive the probabilities related to efficacy and compliance for therapies that are still in clinical trials, we consulted a group of VL experts in an adapted Delphi process to reach consensus after two consultation rounds. In the first round seven clinical experts were presented with a survey asking for efficacy and compliance values for all treatments. Subsequently results from this round were summarized and presented to the experts to revise their earlier answers (round 2). For estimates derived from the literature, we used data from clinical trials using the pooled estimate in the baseline analysis and used the minimum and maximum for sensitivity analysis. All estimates represent definite cure rates defined as the absence of VL at 6 month follow-up; failure, relapse and fatal toxicity are included in the estimates. While minor side effects, such as diarrhoea and vomiting may occur, we did not consider these in the model since they do not hamper the completion of treatment.

**Table 2:** Model parameters.

Variable	Likeliest (base)	Minimum	Maximum	Source
Demographic parameters of sample (%)^b^				
Women in the sample	39	30	50	[18], [34]
Women of childbearing age (15–49 yrs) of all VL	17	10	35	[18], [34]
Patients weighing less than 25 kg	41	20	60	[18], [34]
Children (0–14 years)	51	25	75	[18], [34]
Adults (15–80 years)	49	25	75	[18], [34]
Drug efficacy (%)				
L-AmB + MF	95	90	99	a; [16]
L-AmB + PM	95	90	99	a
MF + PM	95	91	99	a
SSG + PM	90	85	98	a; [34], [35]
MF	94	82	94	[12], [34], [36]–[38]
PM	94	89	95	a; [5], [39]–[41]
AmB	97	96	99	[5], [10], [12], [42]
L-AmB10	95	93	98	a; [15]
L-AmB20	95	93	99	a; [43]
SSG	70	35	93	[1], [2], [34], [39], [41], [44]–[47]
Compliance to treatment (%)				
L-AmB + MF	95	80	97	a
L-AmB + PM	95	85	97	a
MF + PM	95	80	97	a
SSG + PM	83	75	90	a
MF	80	60	90	a; [11]
PM	85	75	90	a
AmB	90	80	90	a
L-AmB10	100	-	-	a
L-AmB20	98	95	100	a
SSG	75	60	90	a
Contraceptive prevalence (%)	55	30	70	a; WHO-WHOSISc

a Estimates obtained from expert opinion (Delphi method).

b Baseline values were varied ±50% in sensitivity analysis.

c Average of figures reported for Bangladesh 58.1% (2004); India 56.3% (2006); Nepal 48.0% (2006).

We assumed treatments of short duration (strategies A, B, C, H and I) to result in high compliance. Similarly, treatment with AmB was assumed to lead to high compliance since treatment is provided on an inpatient basis. On the other hand, patients receiving MF for 28 days receive a 1-week supply of drug at a time for self-administration and compliance is anticipated to be lower than for the other strategies, consistent with findings from a miltefosine phase IV study by Bhattacharya et al (2007) [11] where the final cure rate was 82% on intent-to-treat analysis due to the high losses to follow-up.

### Costs

Table 3 summarizes cost estimates presented in 2008 US dollars (US$). Costs were obtained from primary data collected in 2008 using an ingredients based approach (i.e. collecting information on quantities and prices) and supplemented by data from the literature. We adopted a societal perspective including both provider and patient costs. These costs consist of direct medical costs (e.g. antileishmanial drugs, administration kits (intravenous sets, syringes and needles), laboratory investigations and the cost of hospitalization and outpatient care); direct non-medical costs (transportation to/from the health facility) and indirect costs representing the loss of income to the patient. The cost of drugs was obtained from WHO, Médecins sans Frontières (MSF) and the Institute of One World Health (iOWH). The cost of L-AmB was US$ 20 per 50 mg vial (AmBisome, Gilead, USA), MF (Impavido previously Zentaris, Germany, now Paladin, Canada) US$ 1.41 per 100 mg capsule (or US$ 79 per blister of 56 capsules; the market price of US$ 2.68 per capsule was used as the maximum value in the range), PM US$ 0.71 per 1000 mg ampoule (Gland Pharma Ltd, India), AmB US$ 1.90 per mg vial (Combinopharm, Spain) and SSG US$ 8.25 per vial (Albert David, India). The average drug cost per patient for each strategy was estimated using the anthropometric database. The baseline cost of laboratory investigations includes the cost of equipment, supplies, reagents, the technician's time and indirect laboratory costs (i.e. overhead costs obtained through step-down costing) and is an average cost calculated at a VL treatment centre in India (Muzaffarpur) and a health facility in Nepal (Dharan). The range consists of prices charged to patients at public health facilities and private laboratories. The unit cost per inpatient bed-day and outpatient visit was estimated at a charitable clinic in India [19]. The maximum value used in the range was derived from WHO-CHOICE estimates for the South Asian region [20]. Average income was estimated with the human capital approach and was estimated at US$ 1.48 per day [19]. We assumed that the patient was not able to work for the full duration of treatment. Indirect costs were varied from 0 (i.e. excluding indirect costs) to twice the baseline value in sensitivity analysis. Costs related to diagnosis of VL were not included in the analysis since these are the same for all strategies.

**Table 3:** Cost estimates of each treatment strategy per patient treated (US$ 2008).

Strategy	Drug cost	Other direct medical^1^	Non-medical & indirect	Total cost^2^
L-AmB+MF	95.7	14.8	12.8	123.4
L-AmB+PM	87.1	20.5	25.3	132.9
MF+PM	29.5	19.5	23.8	72.9
SSG+PM	45.1	29.9	43.6	118.6
MF	62.8	22.0	45.4	130.2
PM	14.9	30.6	51.1	96.6
AmB	20.9	131.6	45.4	197.9
L-AmB10	140.0	11.0	2.5	153.4
L-AmB20	280.0	24.7	6.9	311.6
SSG	57.8	40.7	73.4	171.8

1 Includes costs of contraceptives, administration (intravenous kits, solutions, syringes), laboratory investigations. It also includes the cost per inpatient bed-day and outpatient visit obtained.

2 Total costs of strategies with MF in this table do not include cost of AmB given to women in childbearing age that refuse to take contraceptives and are therefore different from total costs mentioned in table 4.

All costs were adjusted to the 2008 national currency of each country using the consumer price index and converted to US dollars using the exchange-rate prevailing at that time.

### Sensitivity analysis

To examine the uncertainty around variables and how these affect the outcome and conclusions of our study, we conducted a series of one-way and two-way sensitivity analyses. Since values for drug efficacy and compliance were largely based on expert opinion we varied the values of these variables over the plausible range specified in table 2. On the cost side, we examined the impact of changing drug prices. While the price of most drugs, such as PM or AmB is unlikely to change much in the future given their low cost, there is uncertainty with regard to the pricing of MF and L-AmB. MF was recently acquired by Paladin Labs Inc., Canada from Zentaris, and it is at the time of writing unclear if the current negotiated differential prices will be maintained. For L-AmB, despite substantial price reductions, the cost per vial remains high and there may be room for further price reductions. To test the robustness of our results we (i) varied each drug cost separately; (ii) conducted a threshold analysis to determine at what price level strategies with L-AmB become the most cost-effective; and (iii) conducted a two-way sensitivity analysis of the price of MF and L-AmB. We also varied the unit cost per inpatient bed-day and outpatient visit. Finally, we examined the impact of indirect costs. In the baseline analysis we assumed the patient would not be able to work for the full duration of treatment. But in practice, with effective treatment, patients may already feel better after a week of treatment and resume their activities. The indirect cost of strategies with longer treatment duration could therefore be overestimated. In addition the inclusion of indirect costs is a controversial issue, mainly due to the valuation method [21]. We therefore looked at the impact of indirect costs by (i) limiting productivity losses to a week, and (ii) excluding indirect costs from estimations.

## Results

### Baseline analysis

Table 4 shows the expected cost and effectiveness for each treatment strategy using baseline values. Strategies were ranked in ascending order of costs. Strategies based on treatment with L-AmB, either used as a single agent or in combination, were found to be more effective compared to other strategies with a single dose of 10mg/kg of L-AmB (strategy H) being the most effective and averting 95% of deaths. This high effectiveness of strategies with L-AmB is explained by the combination of high drug efficacy and a short treatment duration resulting in high expected compliance to treatment. After strategies with L-AmB, the next best alternative is the co-administration of MF and PM (strategy C) averting 90% of deaths. Monotherapies with either SSG (strategy J) or MF (strategy E) had the lowest effectiveness. For SSG this is due to the low efficacy of the drug and for MF due to the low expected treatment compliance.

**Table 4:** Results in the baseline analysis.

Strategy	Cost (C)	Incremental Cost*	Effectiveness (E)	Incremental Effectiveness*	C/E	Incremental C/E (ICER)**
MF + PM	82.5		0.900		92	
PM	96.6	14.1	0.799	−0.101	121	(Dominated)
SSG + PM	118.6	36.1	0.747	−0.153	159	(Dominated)
L-AmB + MF	129.1	46.6	0.942	0.042	137	1123**
L-AmB + PM	132.9	3.8	0.948	0.006	140	652
MF	135.4	2.5	0.761	−0.186	178	(Dominated)
L-AmB 10	153.4	20.6	0.950	0.002	162	8224
SSG	171.8	18.4	0.525	−0.425	327	(Dominated)
AmB	197.9	44.5	0.873	−0.077	227	(Dominated)
L-AmB 20	311.6	158.2	0.949	−0.001	328	(Dominated)

*Numbers in the table are rounded.

**Extended dominance.

The least costly treatment is the co-administration of MF with PM (strategy C) with a cost of $72.9 per patient treated. The cost per patient treated for the other strategies varied from $96.6 (strategy F) to $311.6 (strategy I). The breakdown of costs for each strategy is shown in table 3. The drug cost as a proportion of total costs is the highest for strategies including L-AmB. The price of a 50mg vial of L-AmB at the time of this analysis was $ 20 and is the most expensive VL drug. Obviously, the higher the dosage, the more expensive the treatment. For example the drug cost of strategy I (20mg/kg of L-AmB for 4 days) is $280. The highest “other” direct medical costs were found for strategies requiring prolonged treatment, with treatment on an inpatient basis (strategy G) being the most expensive. Similarly, strategies with long treatment duration and/or requiring many visits to the health facility for administration of the drug have the highest indirect cost.

**Figure 2:**
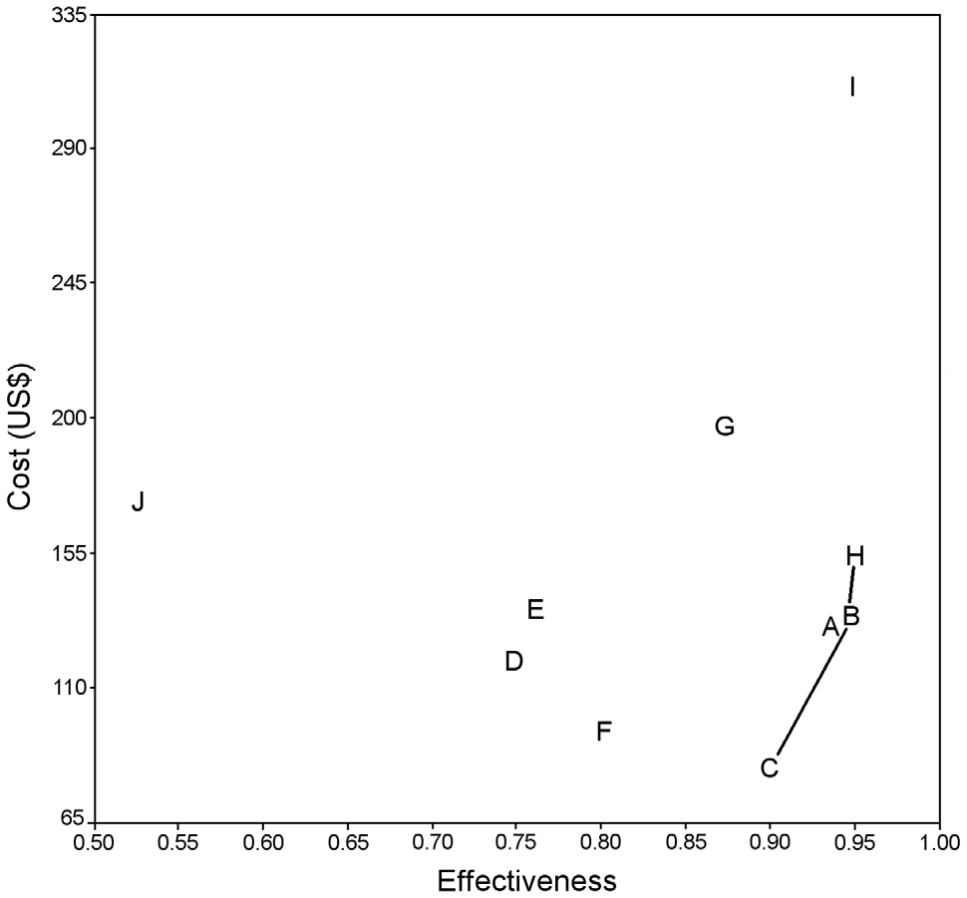
Cost-effectiveness ratio (US$/death averted) of 10 treatment strategies for visceral leishmaniasis. Line CBH shows dominance. All strategies left of this line are dominated by C, B and H, meaning they are equally or less effective and more costly.

Cost-effectiveness results are illustrated in figure 2 and reported in table 4. All treatment strategies on the left of the line are dominated by strategies C, B and H because they are equally or less effective, and either cost more (strong dominance) or have an incremental cost-effectiveness ratio that is higher than the next more effective strategy (extended dominance). The incremental cost, incremental effectiveness, cost-effectiveness ratio (CER) and incremental CER without the dominated strategies are reported in table 5.

**Table 5:** Baseline results without dominated options (simple or extended).

Strategy	Cost	Incremental Cost	Effect	Incremental Effect	C/E	Incremental C/E (ICER)
MF + PM	81.9		0.900		91	
L-AmB + PM	132.9	51.0	0.948	0.047	140	1079
L-AmB10	153.4	20.6	0.950	0.002	162	8224

The most cost-effective strategy appears to be strategy C whereby MF and PM are co-administered. Compared with this strategy, the next most cost-effective strategy is the combination of L-AmB with PM (strategy B), followed by a single dose of 10mg/kg of L-AmB (strategy H). While L-AmB combined with MF (strategy A) is more effective than strategy C, it is also more costly and has a higher (incremental) cost-effectiveness ratio that the next best alternative (i.e. strategy B). In other words the additional cost per death averted is lower for strategy B than strategy A.

### Sensitivity analysis

**Figure 3:**
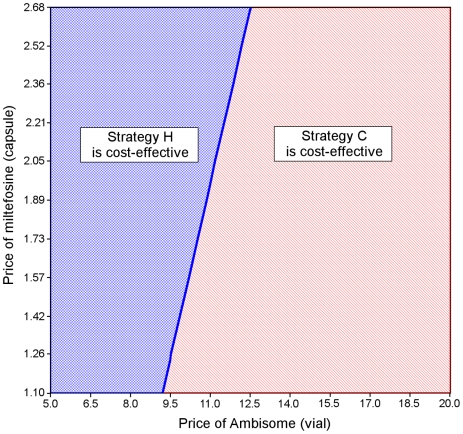
Two-way sensitivity analysis on price of AmBisome and miltefosine.

The study findings were robust to most changes in the input variables. Varying the values of the drug efficacy and compliance over their plausible range did not affect the ranking of strategies. A sensitivity analysis assuming that all non-adherent patients would effectively be cured did not change the ranking of strategies either. With regard to costs, while varying the price of MF did not alter results, the cost-effectiveness results were sensitive to a change in the price of L-AmB. If the price of a vial is decreased by more than 51% to less than $ 9.8, then strategy H becomes the most cost-effective strategy. The relationship between the price of MF and the price of L-AmB and their impact on the ranking of strategies according to their cost-effectiveness, keeping all other variables at their baseline values is shown in figure 3. If MF is purchased at *market price* ($ 2.68 per capsule), the price per vial of L-AmB would need to decrease to below $12.5 for strategy H to become the most cost-effective strategy. Varying the assumptions regarding indirect cost did not change conclusions.

## Discussion

The current first line regimen in the Indian subcontinent is MF for 28 days. There are concerns however that the uncontrolled provision of the drug may increase the likelihood of development of parasite resistance [22]. Even when monitored, patient compliance is not optimal [11], [22] and the risk remains that women in childbearing age receiving MF do not (or only partially) take contraceptives. Our analysis shows that combination therapies for the treatment of VL are a cost-effective alternative to the current strategy in the Indian subcontinent; this finding may be of interest to control programmes regarding the cost-effectiveness of the currently recommended option. The co-administration of MF with PM for 10 days seems to be the most cost-effective option because of the combined effect of low cost, especially drug cost, and high effectiveness. Also, one would expect that the parenteral intramuscular injection of PM by health workers ensures that patients also take the oral MF as it would be directly observed. With the short treatment duration this is likely to result in high patient compliance, increasing the overall effectiveness of the strategy. Although strategies with L-AmB were the most effective, the high drug cost results in a higher average cost-effectiveness. The next best alternative compared to the combination MF/PM was a combination of L-AmB with PM with an incremental cost-effectiveness of $652 per death averted. All other strategies, with the exception of a single dose of 10mg per kg of liposomal amphotericine B were dominated. The relatively poor effectiveness for MF monotherapy in our model is linked to the estimated low adherence when using self-administration with 1-weekly drug supplies. However, alternative drug delivery strategies for MF monotherapy are possible. A strategy where intake of MF would be directly observed would result in significantly higher effectiveness, although at higher direct and indirect costs. When we ran a sensitivity analysis with MF compliance put at 100%, this did not change the ranking of strategies or conclusions.

This study is the most comprehensive cost-effectiveness analysis of alternative strategies for the treatment of VL for the Indian subcontinent to date. We used a simple decision analytical model to compare from a societal perspective the cost and outcome of all possible treatment strategies identified through consultation with experts (Delphi method). The demographic probabilities used in the model, as well as the calculation of the average drug cost was based on real patient data on sex, age and weight obtained from a charitable medical facility in India [23], instead of calculating the drug cost of an “average” 35kg patient as done in other studies. In addition, all cost data in the baseline analysis were based on primary data collected from various sites in Nepal and India. Extensive sensitivity analysis was also done to evaluate the robustness of our estimations and conclusions. The analysis has several limitations. *First* while the use of the anthropometric data can be a strength, the VL treatment centre in Muzaffarpur (Bihar) might not be entirely representative for all VL cases, especially with regard to the male to female ratio. Reassuringly, in a larger series of 4170 patients from two locations in Bihar and 1311 from Nepal the male to female ratio was similar (57∶43, Olliaro et al, manuscript submitted). Although various studies have reported a higher proportion of male patients to be affected by kala-azar [24], [25] there may be under-reporting of kala-azar in women [26] due to sex-selective treatment seeking whereby “fewer women may seek treatment because of its expense” [27]. A M/F ratio in the VL population closer to unity could lower the effectiveness of strategies including MF. *Second*, the drug efficacy estimates for the combination treatments, and the monotherapies with L-AmB were based on input from a Delphi survey of VL experts. The uncertainty surrounding these subjective estimates was minimized by including experts that were clinicians and/or involved in clinical trials of combination treatments and dose-finding studies. The uncertainty was also analysed extensively in sensitivity analysis. *Third* the effectiveness estimates are heavily influenced by the parameters of patient compliance to treatment. Experts assumed treatments with parenteral or intramuscular administration to lead to high compliance and oral treatment to result in lower patient compliance. Given the evidence from the international literature for other diseases, and the limited information available for kala-azar [11], [28], these assumptions seem plausible. As more evidence becomes available from clinical trials (especially Phase IV and other operational studies) and future studies assessing patient compliance, we will update the input parameters and ranges from our model. *Finally*, some cost variables were not included in the analysis. L-AmB requires a cold chain. Because it was difficult to quantify the cost of the cold chain, we did not include it in our calculations and the cost per patient treated and cost per death averted may therefore be an underestimation. There is, moreover, also a substantial risk of breakdowns in the cold chain system, which may impact on the efficacy of the drug. Governments may want to adapt the drug policy choice to the technology constraints in each level of the health system.

Few other studies have investigated the cost-effectiveness of VL treatment strategies. Vanlerberghe et al (2007) [29] compared various monotherapies from a health service perspective (not including paromomycin) and found a strategy with miltefosine to be the most cost-effective with US$328 per death averted. Although the study uses a similar decision tree model and sensitivity analysis to account for uncertainty in the input parameters, the results from this study cannot be compared with ours. The model by Vanlerberghe et al. starts with a clinical suspect going through diagnosis and then treatment. Treatment effectiveness is therefore defined by probabilities other than those directly related to treatment such as the prior probability of disease, and the sensitivity and specificity of the diagnostic test. Patient compliance was not modelled either. A more recent study by Olliaro et al. (2009) [30] compared various monotherapies and a combination of L-AmB with MF with different total dosages for MF from a health systems perspective. Similar to our findings, Olliaro et al show that the combination L-AmB+MF (for 8 days) with a cost of $124–160 per death averted is more cost-effective than most monotherapies (the exception being PM delivered in an outpatient setting and a 5mg/kg single dose formulation of L-AmB). However this study did not include indirect costs (i.e. productivity losses) underestimating the effect of strategies with a short treatment duration that are beneficial to the patient and household.

Our results highlight that several possible therapeutic options may exist for the South Asian context - especially in light of the ongoing VL elimination campaign in the Indian subcontinent - but combination regimens are efficient options compared to monotherapy. The analysis should be repeated in other VL-endemic areas such as East Africa and Brazil where efficacy outcomes, treatment regimens, direct and indirect costs may differ considerably. Critical elements of importance to national and international policy makers are the cost of drugs, the level of out-of-pocket expenditures by VL patients and compliance to treatment. An obstacle to the introduction of strategies with L-AmB in national control programmes is the cost of the drug. Despite substantial reductions in the price of AmBisome over the past years (more recently to $18 for a 50mg vial), the threshold analysis showed this to be not enough to make strategies with L-AmB a cost-effective alternative. In addition, the capacity of VL patients and their family to cover the costs of treatment is very limited. VL is a disease that affects the poorest of the poor [31] and places a considerable economic burden on households [19], [32], [33]. Especially in India and Bangladesh, the combination of frequent drug shortages and poor quality of care in public health facilities pushes many patients to buy drugs from private pharmacies or to seek care in the private sector. Unless the government or a donation programme covers the cost of drugs, strategies including expensive drugs such as L-AmB will be a barrier to patients and reduce access to appropriate and effective care resulting in increased mortality.
